# Combined Application of Aminoglycosides and Ascorbic Acid in the Elimination of *Proteus mirabilis* Rods Responsible for Causing Catheter-Associated Urinary Tract Infections (CAUTIs)—A Molecular Approach

**DOI:** 10.3390/ijms232113069

**Published:** 2022-10-28

**Authors:** Paulina Stolarek, Przemysław Bernat, Antoni Różalski

**Affiliations:** 1 Department of Biology of Bacteria, Faculty of Biology and Environmental Protection, University of Lodz, Banacha 12/16, 90-237 Lodz, Poland; 2 Department of Industrial Microbiology and Biotechnology, Faculty of Biology and Environmental Protection, University of Lodz, Banacha 12/16, 90-237 Lodz, Poland

**Keywords:** *Proteus mirabilis*, adhesion, catheter, aminoglycosides, ascorbic acid, fatty acids, phospholipids, membrane permeability, hydroxyl radical

## Abstract

*Proteus mirabilis* is a common cause of catheter-associated urinary tract infections (CAUTIs). In this study, we verified the effectiveness of amikacin or gentamicin and ascorbic acid (AA) co-therapy in eliminating uropathogenic cells, as well as searched for the molecular basis of AA activity by applying chromatographic and fluorescent techniques. Under simulated physiological conditions, a combined activity of the antibiotic and AA supported the growth (threefold) of the *P. mirabilis* C12 strain, but reduced catheter colonization (≤30%) in comparison to the drug monotherapy. Slight modifications in the phospholipid and fatty acid profiles, as well as limited (≤62%) 2’,7’-dichlorofluorescein fluorescence, corresponding to the hydroxyl radical level, allowed for the exclusion of the hypothesis that the anti-biofilm effect of AA was related to membrane perturbations of the C12 strain. However, the reduced (≤20%) fluorescence intensity of propidium iodide, as a result of a decrease in membrane permeability, may be evidence of *P. mirabilis* cell defense against AA activity. Quantitative analyses of ascorbic acid over time with a simultaneous measurement of the pH values proved that AA can be an effective urine acidifier, provided that it is devoid of the presence of urease-positive cells. Therefore, it could be useful in a prevention of recurrent CAUTIs, rather than in their treatment.

## 1. Introduction

*Proteus mirabilis*, an opportunistic pathogen belonging to the *Morganellaceae* family, is a common cause of urinary tract infections (UTIs), especially those detected in patients with urinary tract structural or neurological abnormalities [1]. One of them is the neurogenic bladder defined as bladder dysfunction resulting from a central or peripheral neurologic insult [2]. The incidence of *Proteus* rods in the neurogenic population amounts to a small percentage [3]. However, catheter-associated UTIs (CAUTIs) mediated by *P. mirabilis* are particularly frequent (10–44%) and pose a threat to the health and even life of long-term-catheterized patients [4]. Crystalized biofilms may lead to the incrustation of a catheter and its obstruction blocking the flow of urine, which might result in urinary retention in the bladder, bacteriuria episodes, fever, sepsis, and shock [1]. On the other hand, the biofilm structure limits penetration and distribution of drugs to the bacteria located in its deeper layers, enabling further bacteria multiplication despite the presence of antibiotics [5]. Therefore, complicated UTIs, caused by biofilm-forming *P. mirabilis*, pose increasing medical challenges.

Antibiotics most commonly applied in UTI treatment include trimethoprim, fluoroquinolones, β-lactams, nitrofurantoin, fosfomycin, and trimethoprim-sulfamethoxazole, of which the last three are guideline-recommended first-line agents [6]. Between 2002 and 2012, an enhanced resistance of *P. mirabilis* to cefotaxime, ceftazidime, and ciprofloxacin was observed [7]. Similarly, the increased prevalence of resistance of other uropathogens to amoxicillin, ampicillin, cotrimoxazol, first-generation cephalosporins, and amoxicillin-clavulanic acid has been reported, suggesting that second- and third-generation cephalosporins, fosfomycin, and aminoglycosides should be used in UTI therapy [8,9]. Amikacin seems to be more effective against *P. mirabilis* than gentamicin [10].

Aminoglycosides are well-known drugs that impair bacterial protein synthesis through binding to the 30S ribosomal subunit. These antibiotics are highly potent, broad-spectrum drugs with many desirable properties to treat serious Gram-negative bacterial infections [11]. Aminoglycosides are an ideal drug class for single-dose treatment of UTIs due to their excretion to urine in high concentrations (400 µg mL^−1^ of gentamicin in urine after a single dose of 1 mg kg^−1^, i.e., 100-fold of breakpoint for *Enterobacteriaceae*) and their remaining in the urinary tract at amounts above the therapeutic levels for 72 h or longer (for most uropathogens) [12]. Unfortunately, the use of aminoglycoside antibiotics has its side effects, such as nephrotoxicity, hearing loss, vestibular toxicity, and neuromuscular blockade. However, these side effects are typically dose dependent and are observed in patients who receive high doses of aminoglycosides for a protracted period [12].

Ascorbic acid (AA) is often recommended as a prophylactic agent for prevention of recurrent urinary tract infections. Research on the effectiveness of AA supplementation has been carried out on diverse groups including healthy volunteers [13], pregnant women [14], children with febrile upper urinary tract infection [15], spinal cord injury patients [16], calcium-stone-forming patients [17], and kidney transplant patients [18]. The published results are contradictory. Due to a lack of strong clinical evidence, the role of ascorbic acid as a urine acidifier still remains controversial. Interestingly, there are also reports on antibacterial [19,20] and anti-biofilm [21,22,23,24] properties of ascorbic acid. However, the mechanisms of its action are still poorly understood.

Taking into account the above information, we hypothesized that the application of combined treatment with ascorbic acid and an aminoglycoside may bring benefits, such as limitation of the applied dose of the antibiotic, side effects of its action, and hospitalization time, to patients with UTIs and CAUTIs caused by *P. mirabilis*.

In this study, the effectiveness of combined treatment with amikacin (AK) or gentamicin (CN) and AA (compared to the monotherapies) in the limitation of both the growth of *P. mirabilis* and its ability to colonize the Foley catheter was investigated. The efficiency of AA in the bacterial culture acidification was verified in a multifaceted manner—by measuring the pH value, quantitative determination of ascorbic acid concentration, and evaluation of the activity of urease as a key enzyme in urine alkalization. In an attempt to explain the molecular basis of AA activity in combined treatment with aminoglycosides, its influence on the *P. mirabilis* membranes was examined. Qualitative and quantitative analyses of phospholipids (PLs) and fatty acids (FAs), dynamic membrane components being probable biomarkers of toxicity or drug resistance, were performed using high–performance liquid chromatography (HPLC) and gas chromatography (GC) techniques coupled with mass spectrometry (MS). Hydroxyl radical (HO•) production and cell membrane permeability changes, generated by both aminoglycosides and ascorbic acid, were investigated using high fluorescent probes: propidium iodide (PI) and 2’,7’-dichlorofluorescein (DCF).

## 2. Results

### 2.1. Bacterial Growth and Catheter Colonization by the P. mirabilis Cells in the Presence of Aminoglycosides and/or Ascorbic Acid

**Figure 1:**
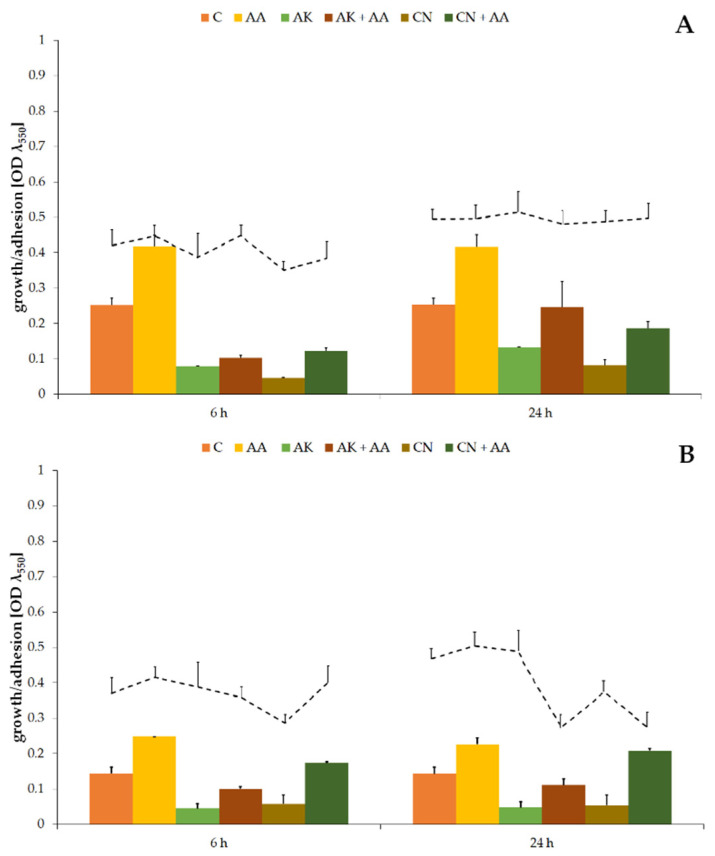
The bacterial growth (column graphs) and cell adhesion to the catheter (line graphs) of the *P. mirabilis* ATCC 29906 (**A**) and C12 (**B**) strains after 6 and 24 h of cultivation. Legend: C—control, AA—ascorbic acid, AK—amikacin, CN—gentamicin.

The tested substances had a statistically significant (*p* ≤ 0.05) influence on the quantity of *P. mirabilis* cells, as well as their ability to colonize the catheter surface (column graphs on Figure 1).

Optical densities of non-treated cultures were 0.253 ± 0.019 (Figure 1A) and 0.143 ± 0.018 (Figure 1B) for the ATCC 29906 and C12 strains, respectively. A treatment with high concentrations of amikacin or gentamicin resulted in a several-fold decrease (*p* ≤ 0.01) in the OD values. Reduction in the number of uropathogenic cells, but not their elimination, indicates resistance of the *P. mirabilis* strains to the tested antibiotics. Ascorbic acid treatment brought about results opposite to those expected, i.e., it supported uropathogenic cell multiplication by at least 58% compared to the non-treated cells. Moreover, combined activity of AA and AK or CN resulted in even several times higher (*p* ≤ 0.01) OD values in comparison to aminoglycoside monotherapies.

In contrast to the evident influence of the tested substances on the number of *P. mirabilis* planktonic cells, their impact on the quantity of uropathogenic adherent cells was not so obvious (line graphs on Figure 1). During the first 6 h of gentamicin treatment, the ATCC 29906 and C12 strain adhesion to the catheter surface was limited by 20%, but after 24 h, the degree of catheter colonization was similar to that observed in the non-treated cultures. On the other hand, after 24 h combined activity of the aminoglycosides and AA, 38% and 22% reductions, respectively, of the catheter cover by the C12 cells in comparison to AK and CN monotherapies were noticed.

### 2.2. Concentration and Stability of the Tested Substances during the Experiments

In order to verify the stability of aqueous solutions of the aminoglycosides and ascorbic acid as well as to quantify the substances used in experiments, the analyses involving a HPLC–MS^2^ technique were performed. The obtained results are presented in Table 1.

First of all, high stability of the aminoglycoside solutions throughout the research was confirmed. Both the amikacin and gentamicin levels oscillated around their initial concentrations, i.e., 563 mg L^−1^ for AK and 600 mg L^−1^ for CN. On the contrary, the high stability of the ascorbic acid solution could not be shown. Its concentration decreased progressively, amounting to 0.02 g L^−1^ ± 0.01 g L^−1^ after 24 h. Moreover, in the presence of uropathogenic cells, AA was rapidly lost, being close to the detection limit.

### 2.3. Changes of pH Values in P. mirabilis Cultures Supplemented with AA

Due to the common opinion about ascorbic acid as a urinary acidifying agent, its influence on medium pH values was studied. Although AA initially acidified the TSB medium to a pH below 6 (column graphs in Figure S1), its value exceeded 8 in all *P. mirabilis* cultures immediately following 6 h of the experiment.

Quick alkalization of the growth environment could have been the result of utilization of urea, a substrate for urease, that was readily available in the modified TSB medium (9.3 g L^−1^). Therefore, ammonia excretion by the uropathogens was examined. Increased levels of ammonia (line graphs in Figure S1) corresponded to heightened culture pH values. Despite the limited *P. mirabilis* growth caused by the urea presence (data not shown), alkalization of the cultures was associated with cell metabolism, as evidenced by the constant pH values in time (5.39 ± 0.19) of the AA abiotic controls (data not shown).

### 2.4. Effect of Aminoglycosides and Ascorbic Acid on the P. mirabilis Urease Activity

Although the ineffectiveness of AA as a urinary acidifier had been proven in the previous experiment, we decided to verify the way in which the aminoglycosides and ascorbic acid affect the activity of urease, a key enzyme for urine alkalization. The obtained results are presented in Figure S2.

The urease activity amounted to 100.47 U ± 9.76 U (Figure S2A) and 85.87 U ± 8.65 U (Figure S2B) in the ATCC 29906 and C12 cells, respectively, and it increased over time. A significant influence of AA treatment on urease activity (*p* ≤ 0.05) was observed in the 6 h ATCC 29906 cells. Ascorbic acid supplementation resulted in a 21% decrease in the enzyme activity. On the other hand, combined activity of AA and AK or CN stimulated urease activity, increasing the level of excreted ammonia by about 20%. A similar effect was noticed for the C12 cells treated with a mixture of AA and AK.

### 2.5. Modification of the P. mirabilis Phospholipid Profile Induced by AA, AK, or CN Treatment

Due to the fact that before binding to the ribosome, aminoglycosides have to cross the membrane barrier, it was decided to investigate PLs, crucial membrane components. The results of liquid chromatographic separation and identification of molecules by tandem mass spectrometry are shown below.

**Figure 2:**
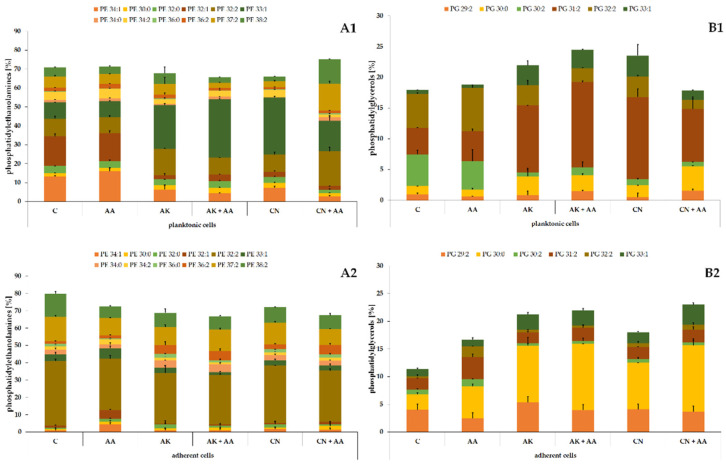
Percentage content of phosphatidylethanolamines (**A**) and phosphatidylglycerols (**B**) identified in the planktonic (**1**) and adherent (**2**) cells of the *P. mirabilis* ATCC 29906 strain treated with ascorbic acid, amikacin, or gentamicin. Legend: C—control, AA—ascorbic acid, AK—amikacin, CN—gentamicin.

The phospholipid profile of the ATCC 29906 cells (Figure 2) consisted mostly of (≈70%) phosphatidylethanolamines (PE), among which PE 34:1, PE 32:1, PE 32:2, and PE 33:1 were dominant in the planktonic cells, whereas PE 32:2, PE 37:2, and PE 38:2 were the main lipids in the adherent cells. Of the remaining (≈30%) phosphatidylglycerols (PG), PG 30:2, PG 31:2, and PG 32:2 had the highest levels in the planktonic cells, while PG 29:2, PG 30:0, and PG 31:2 were the most abundant in the adherent cells.

**Figure 3:**
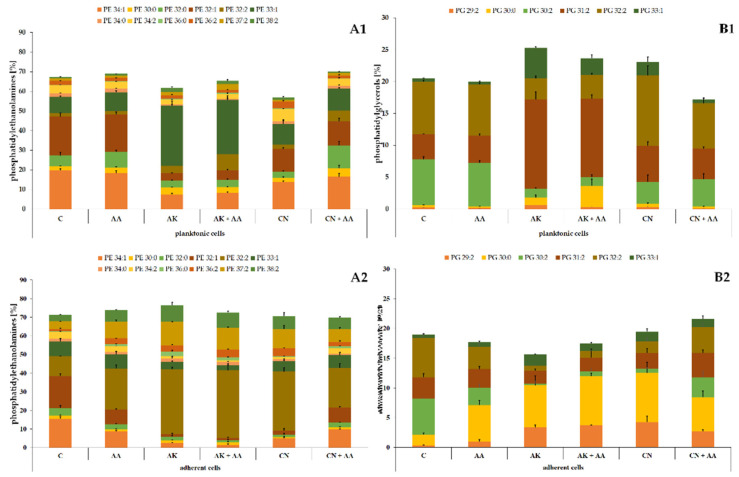
Percentage content of phosphatidylethanolamines (**A**) and phosphatidylglycerols (**B**) identified in the planktonic (**1**) and adherent (**2**) cells of the *P. mirabilis* C12 strain treated with ascorbic acid, amikacin, or gentamicin. Legend: C—control, AA—ascorbic acid, AK—amikacin, CN—gentamicin.

Similarly to the ATCC 29906 cells, the C12 cells were composed of PEs and PGs in the ratio of approximately 70% to 30% (Figure 3). Quantitatively, PE 34:1 followed by PE 32:1, PE 33:1, PG 32:2, and PG 30:2 had the highest levels in the planktonic cells. In addition to the above, PE 32:2 was also one of the most abundant PLs in the adherent cells. The variety in the phospholipid composition of planktonic and adherent cells was discussed in detail in our previous work [25].

The significance of changes in the *P. mirabilis* phospholipid composition induced by the aminoglycosides or ascorbic acid treatment was verified using Student’s *t*-test. The results of the statistical analyses are presented in Table 2.

The ascorbic acid treatment caused the least noticeable modifications in the PL profile of the uropathogens. Quite the opposite, the lipid composition of the bacterial cells was strongly changed by the aminoglycoside action. AK and CN acted similarly on the ATCC 29906 and C12 planktonic cells, decreasing the levels of the following lipids: PE 34:1 (≤84%), PE 32:0 (≤40%), PE 32:1 (≤87%), PE 34:0 (≤80%), and PG 30:2 (≤87%) (marked by * on the green background in Table 2), and increasing the contents of PE 33:1 (≤3.5-fold), PE 36:0 (≤3.4-fold), PG 30:0 (≤3.2-fold), PG 31:2 (≤3.5-fold), and PG 33:1 (≤9-fold) (marked by * on the red background in Table 2).

Moreover, the aminoglycosides had a similar effect on the bacterial adherent cells, causing a decrease in the two following PLs levels: PE 32:1 (≤92%) and PG 30:2 (≤88%), and an increase in the amounts of PE 36:0 (≤10.5-fold), PE 36:2 (≤3.5-fold), PG 30:0 (≤4.5-fold), and PG 33:1 (≤3-fold) (marked by ** on the green background in Table 2). As described above, the phospholipid profiles of the uropathogens treated with the antibiotics were drastically altered, which indicates the aggressive action of these compounds against biological membranes, or, quite the opposite, adaptive changes of the *P. mirabilis* membranes associated with the resistance mechanisms against the aminoglycosides.

Student’s *t*-test was also used to determine significant modifications in the *P. mirabilis* PL profile caused by combined treatment with ascorbic acid and amikacin or gentamicin, compared to aminoglycoside monotherapies. The results of the statistical analyses are presented in Table 3.

In contrast to the easily noticeable common pattern in aminoglycoside-induced quantitative changes in the bacterial PLs, such a scheme for modifications of lipids induced by the combined action of the antibiotics and ascorbic acid was not observed. The applications of AA and AK mixture resulted in higher PE 32:1 (≤68%) and lower PE 38:2 (≤47%) levels, respectively, while the presence of AA with CN caused decreased the amounts of PE 34:2 (≤61%), PG 32:2 (≤55%), and PG 33:1 (≤73%) and increased amounts of PE 32:2 (≤2.5-fold) and PE 37:2 (≤4.5-fold) in the bacterial planktonic cells, compared to the results obtained using the monotherapies (marked by * in Table 3).

For the adherent cells, these dependencies were as follows: supplementation with ascorbic acid increased the quantity of PG 30:2 (≤2.5-fold) and decreased the levels of PE 32:0 (≤53%), PE 33:1 (≤52%), PE 34:2 (≤35%), and PE 36:0 (≤45%) in comparison to the results obtained for the AK treatment. On the other hand, the presence of AA resulted in higher amounts of PE 30:0 (≤2-fold), PE 32:1 (≤3.7-fold), and PG 32:2 (≤2.2-fold) and lower abundance of PE 37:2 (≤33%) compared to the results obtained for the CN treatment (marked by ** in Table 3).

Due to more white spaces being present in Table 3 than in Table 2, it can be deduced that the application of AA in combined treatment had a weak effect on the strongly antibiotic-altered PL profiles of the uropathogens.

### 2.6. Quantitative Changes in Fatty Acids of the Uropathogenic Cells Caused by the Antibiotics and/or Ascorbic Acid Presence

**Figure 4:**
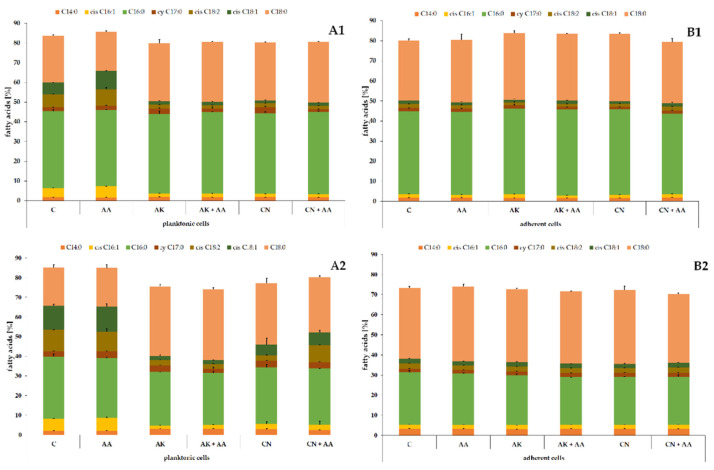
Percentage contents of FAs identified in the planktonic (**A**) and adherent (**B**) cells of the ATCC 29906 (**1**) and C12 (**2**) strains treated with ascorbic acid and/or aminoglycosides. Legend: C—control, AA—ascorbic acid, AK—amikacin, CN—gentamicin.

In the next stage, lipids remaining after HPLC–MS^2^ analyses were transformed to fatty acid methyl esters and determined using the GC–MS method. The obtained FAs, as potential indicators of toxicity of compounds disturbing the membrane structure, are shown in Figure 4.

The fatty acid profile consisted of saturated FAs (myristic, C14:0; palmitic, C16:0; and stearic, C18:0), monounsaturated FAs (palmitoleic, *cis* C16:1, and oleic, *cis* C18:1), one polyunsaturated FA (linoleic, *cis* C18:2), and one alicyclic FA (*cis*-9,10-methylenehexadecanoic acid, *cy* C17:0). Palmitic and stearic acids constituted the majority of the total FA content in the *P. mirabilis* cells. The cumulative content of these two fatty acids ranged from 52% in the C12 planktonic cells to 72% in the ATCC 29906 adherent cells. The differences in the fatty acid composition of the planktonic and adherent cells was described in detail in our previous paper [25].

To indicate significant modifications in the *P. mirabilis* FA profile caused by ascorbic acid, amikacin, or gentamicin treatment, Fisher’s test was applied. The results of the statistical analyses are presented in Table 4.

Unlike the ascorbic acid treatment, the aminoglycoside action induced considerable changes in the FA profile of the *P. mirabilis* cells. AK and CN had a similar effect on the planktonic cells of the ATCC 29906 and C12 strains. The increased level of *cy* C17:0 (≤60%) as well as decreased quantities of *cis* C16:1 (≤72%), *cis* C18:2 (≤76%), and *cis* C18:1 (≤83%) were noted in the uropathogenic cells treated with the antibiotics (marked by * in Table 4). Unfortunately, due to the lack of noticeable aminoglycoside-induced changes in the FA profile of the adherent C12 cells, indication of a common pattern in fatty acid quantitative changes in both examined strains became impossible. Therefore, the effect of AK and CN on the *P. mirabilis* FA profile could be defined as strong for the planktonic cells and weak for the adherent cells.

Fisher’s test was also used to determine significant modifications in the *P. mirabilis* FA profile induced by combined treatment with ascorbic acid and amikacin or gentamicin, compared to the aminoglycoside monotherapies. The results of the statistical analyses are presented in Table 5. Despite significant changes in the amount of several FAs (green and red spaces), no common trend in quantitative fatty acids changes in either planktonic or adherent *P. mirabilis* cells was established.

### 2.7. Quantification of Reactive Oxygen Species in the Bacterial Cells Exposed to the Antibiotics and/or Ascorbic Acid

**Figure 5:**
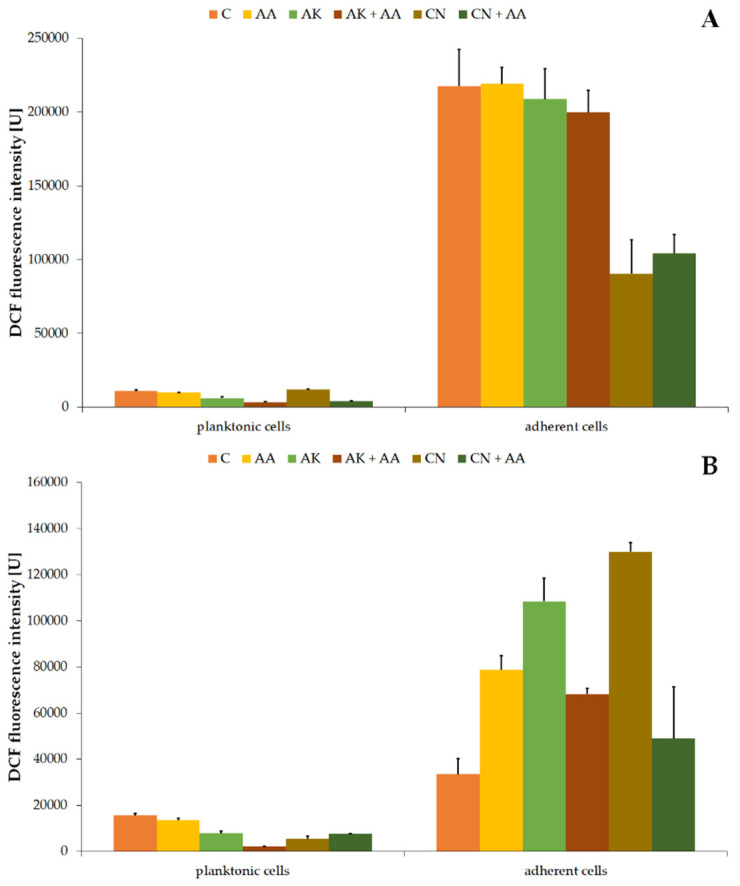
Changes of 2’,7’-dichlorofluorescein (DCF) fluorescence intensity in the planktonic and adherent cells of the ATCC 29906 (**A**) and C12 (**B**) strains treated with AA, AK, or CN. Legend: C—control, AA—ascorbic acid, AK—amikacin, CN—gentamicin.

It is known that aminoglycosides and ascorbic acid supply a substrate for the Fenton reaction, which results in hydroxyl radical formation. The level of these free radicals, damaging DNA, lipids, and proteins, was estimated through measurement of DCF fluorescence intensity. The obtained results are presented in Figure 5.

The values of DCF fluorescence intensity in the non-treated *P. mirabilis* planktonic cells were 10,929.97 U ± 797.14 U (Figure 5A) and 15,521.06 U ± 908.33 U (Figure 5B) for the ATCC 29906 and C12 strains, respectively. AA treatment had no significant effect on the hydroxyl radical level, while AK treatment limited its amount by at least 40%. The combined activity of amikacin and ascorbic acid lowered the DCF fluorescence intensity by at least 44%, compared to its value in the antibiotic monotherapy. On the other hand, the combined activity of gentamicin with ascorbic acid resulted in a 40% enhancement of the red fluorescence intensity in comparison to the drug monotherapy.

Higher levels of intracellular HO• were found in the adherent cells than in the planktonic cells. Fluorescence values of the used fluorescein were 217,365.19 U ± 24,891.91 U (Figure 5A) and 33,491.55 U ± 6744.61 U (Figure 5B) in the non-treated adherent ATCC 29906 and C12 cells, respectively. Neither ascorbic acid nor amikacin had a significant effect (*p* ≥ 0.05) on the hydroxyl radical production in the ATCC 29906 cells. On the other hand, all tested substances stimulated (≤3.9-fold) the HO• formation in the C12 cells, while combined treatment with ascorbic acid and antibiotics reduced the free radical production (≤62%), compared to the antibiotic monotherapies.

### 2.8. Influence of AA, AK, or CN on the P. mirabilis Membrane Permeability

**Figure 6:**
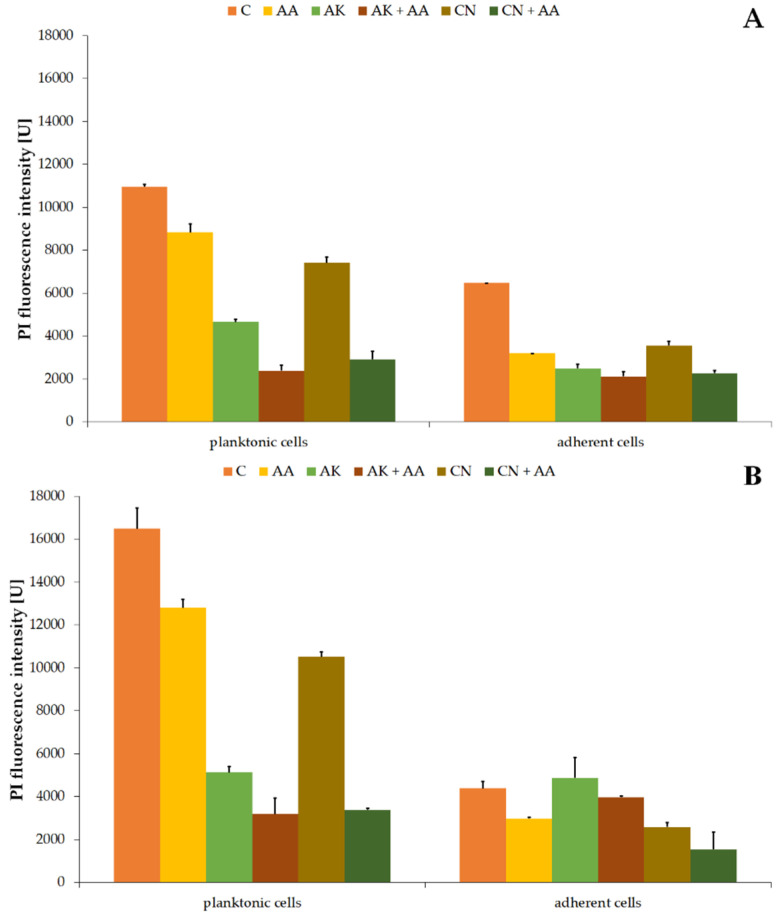
Changes of propidium iodide (PI) fluorescence intensity in the planktonic and adherent cells of the ATCC 29906 (**A**) and C12 (**B**) strains treated with AA, AK, or CN. Legend: C—control, AA—ascorbic acid, AK—amikacin, CN—gentamicin.

Due to drastic, aminoglycoside-induced changes in the lipid composition of the examined *P. mirabilis* cells, we hypothesized about the disturbed membrane permeability of the uropathogens. This hypothesis was verified by measuring the red fluorescence intensity of propidium iodide, which entered the cells and was intercalated to bacterial DNA. Enhanced intensity of PI fluorescence was regarded as an increment of the cell membrane permeability. The obtained results are shown in Figure 6.

PI fluorescence intensity values in non-treated planktonic cells were 10,953.94 U ± 111.75 U (Figure 6A) and 16,486.57 U ± 952.23 U (Figure 6B) for the ATCC 29906 and C12 strains, respectively. Applied therapies resulted in significantly weakened red fluorescence. Membrane permeability for propidium iodide decreased by at least 20%, 60%, and 30% in the AA-, AK-, and CN-treated cells, respectively. Moreover, the combined activity of AA and the aminoglycosides lowered the PI fluorescence intensity even more (by 40% and 60%) than AK and CN monotherapies, respectively.

Red fluorescence intensity values in non-treated adherent cells were 6468.73 U ± 5.75 U (Figure 6A) for the ATCC 29906 strain and 4389.77 ± 308.69 U (Figure 6B) for the C12 strain. In the AA-treated cells, at least 32% reduction (*p* ≤ 0.05) of PI fluorescence intensity was noted. In turn, the amikacin-altered permeability of the cell membrane for PI decreased by at least 20% (*p* ≤ 0.05). Ascorbic acid supplementation in combined treatment limited membrane permeability even more (≈60%) than in the CN monotherapy.

## 3. Discussion

Spreading bacterial resistance coupled with the advantages of the UTI treatment with aminoglycosides contributed to the resurgence of clinicians dealing with the therapeutic potential of these antibiotics. Recently, Goodlet et al. [12] confirmed 94.5% effectiveness of single-dose aminoglycoside treatment for 471 UTI patients infected by *E. coli*, *Proteus* spp., and *Klebsiella* spp. In the past 10 years, high activities of amikacin and gentamicin against *P. mirabilis* planktonic cells were frequently reported [7,10,26,27]. Our studies also showed a limitation of the ATCC 29906 and C12 planktonic cells growth during aminoglycoside treatment. Although the applied concentrations exceeded EUCAST clinical breakpoints (35-fold for AK and 150-fold for CN), the uropathogenic cells were not completely eliminated, which indicated the resistance of the ATCC 29906 and C12 strains to aminoglycoside antibiotics.

Bacterial biofilms are complicated structures composed of bacteria surrounded by a matrix of extracellular polymeric substances, acting as a barrier for drug penetration. Therefore, the production of bacterial biofilms is considered as one of the main causes of bacterial resistance and of difficulties in treating biofilm-related infections [28]. *P. mirabilis* is known for its ability to grow in biofilms on abiotic (e.g., silicone, polystyrene, latex, glass) and biological surfaces. However, *P. mirabilis* biofilms developed in the urinary tract, especially those on the catheters surface, are the most extensively examined [5].

Our research focused on the first stage of biofilm formation, i.e., adhesion of uropathogens to a silicone catheter surface. Gentamicin treatment limited the adhesion of the *P. mirabilis* cells to the catheter. However, after 24 h, despite the constant 600 mg L^−1^ concentration of the antibiotic, the catheter colonization by the ATCC 29906 cells was advanced, similarly to that observed in the non-treated cultures. On the other hand, treatment with amikacin had no effect on reducing the colonization of the catheter surface by the ATCC 29906 and C12 cells. Resistance of biofilm-forming clinical isolates of *P. mirabilis* to aminoglycosides was noticed. Sahal and Bilkay [29] showed that below 20% of the 15 tested *P. mirabilis* strains, which were capable of biofilm formation, were resistant to CN. Subsequently, Shaaban et al. [30] pointed to four out of seven *P. mirabilis* isolates that were resistant to amikacin. Extended research of Vašková et al. [31] on the activity of gentamicin on sessile cells of uropathogenic *E. coli*, *Klebsiella* spp., *P. mirabilis*, and *P. aeruginosa* clinical isolates led to the estimation of the minimal biofilm eradicating concentrations of CN, which were in the range from 8 to 512 mg L^−1^ depending on the strain. On the other hand, Wasfi et al. [32] tested the effect of the sub-MIC levels of gentamicin on the adherence of clinical *P. mirabilis* strains to a micro-plate. Up to 70% reduction in biofilm formation was achieved using a concentration of CN corresponding to ½ MIC. However, due to the fact that the concentrations applied in the studies were not presented in the paper, the sensitivity of these strains to anti-adhesive action of gentamicin is difficult to assess. On the other hand, in a paper published by Moryl et al. [26], the authors reported that only 5% (from 50 isolates) of sessile *P. mirabilis* forms were susceptible to the effects of amikacin. In other studies, AK reduced the biofilm viability of two out of the four tested *P. mirabilis* strains (even about 54% ± 15% for C41 isolate) [33] or even all tested 15 uropathogenic strains, completely eradicating their biofilms [29]. In contrast to the considerable antibacterial effectiveness of aminoglycosides against *P. mirabilis* planktonic forms, their monotherapies were less often successful in eliminating the uropathogenic sessile cells. In most of the above-cited articles, decreased sensitivity to AK or CN treatment for *P. mirabilis* strains forming strong or at least moderate biofilms was noted. The impeded penetration of drugs through the biofilm structure was found to cause up to 1000 times greater resistance to antibiotics of bacteria grown in biofilms than planktonic bacteria [5].

Ascorbic acid supplementation, as a non-antibiotic prophylaxis for UTIs, has both supporters and opponents, and still remains controversial. Antimicrobial activity of AA was demonstrated on uropathogenic *E. coli*, *K. pneumonia*, *P. aeruginosa*, and *P. mirabilis* [19,20]. According to Verghese et al. [20], ascorbic acid treatment with 10 mg mL^−1^ dose resulted in a threefold reduction in the optical densities of both cultures of *E. coli* (from 0.295 ± 0.092 to 0.108 ± 0.014) and *K. pneumonia* (from 0.422 ± 0.085 to 0.113 ± 0.026) on TSB medium. The same AA dose caused growth inhibition zones of *P. mirabilis* (20.7 mm ± 0.8 mm) and *P. aeruginosa* (13.3 mm ± 0.8 mm) on NB medium with agar [19]. Although both studies concluded that ascorbic acid is an effective antibacterial agent, it is important to pay attention to the applied AA concentrations, which exceeded those achievable in urine after oral vitamin C supplementation. Moreover, the experiments were conducted using growth media devoid of urea, an important component of urine. Too high AA concentration and lack of urea could not mimic the physiological conditions during urinary tract infections, which raises doubts about the practical use of the results obtained by Mumtaz et al. [19] and Verghese et al. [20].

The dispute over ascorbic acid as an anti-biofilm agent has remained unresolved, mainly because of unknown mechanisms of ascorbic acid action. However, recently, data have emerged regarding the destabilization of biofilms of uropathogenic *E. coli* UTI89 [21], *E. coli* EMC17 [22], and carbapenem-resistant hypervirulent *K. pneumoniae* [23] by AA through reducing the synthesis of extracellular polymers. Hypothetically, the disruption of the exopolymer matrix by AA would facilitate the treatment of bacterial-biofilm-related infections. Unfortunately, in the available literature, very little information can be found on the action of ascorbic acid during the treatment of *P. mirabilis*-induced CAUTIs. El-Gebaly et al. [24] conducted research on the effectiveness of AA in the eradication of biofilms produced by *Proteus* sp. on catheter surfaces. Ascorbic acid applied in the concentration of 80 mg mL^−1^ caused a reduction in the initial adherence of the *Proteus* sp. cells to catheters by 65%. On the other hand, Kwiecińska-Piróg et al. [34] observed no significant effect of 0.4 mg mL^−1^ ascorbic acid on biofilm formation by any of the five tested *P. mirabilis* strains. In our study, ascorbic acid applied in the intermediate concentration (3.4 g L^−1^) also had a marginal impact on the ATCC 29906 and C12 cells adhesion to the catheter surface. Taking into account the above data, the dose of ascorbic acid seems to be key in the interpretation of the presented results. Too low a concentration might not provide an inhibitory effect, while a much higher one turned out to be effective yet physiologically unachievable.

In our study, the concentration of ascorbic acid that is achievable in the urinary tract [35] and the minimum concentration of urea in urine [36] were used. AA presence intensified the growth of *P. mirabilis* planktonic cells compared to the non-treated cells, disproving preliminary hypothesis about ascorbic acid as an alternative way to treat UTIs. The most probable explanation for the obtained results is utilization of ascorbic acid by the *P. mirabilis* cells as an additional carbon source, which is evidenced by a more rapid loss of the compound in bacterial cultures than in abiotic controls. Many bacteria, including *E. coli*, *K. pneumonia*, *E. faecalis*, and *S. pneumonia*, have been shown to degrade AA, most often via the ascorbate-specific phosphotransferase systems coded by the *ula* operon or its homologs [37,38,39,40,41]. However, no pathways (especially aerobic) of AA assimilation by *P. mirabilis* cells have been described in the available literature. Moreover, as a result of another variant of experiment performed analogously to that described in Section 4.2 but using a TSB medium without urea, a 30% limitation of the *P. mirabilis* ATCC 29906 and C12 cells growth was obtained (data not shown). Therefore, we suppose that the presence of urea in the samples could play a crucial role in the discrepancy of results published by different authors. Unfortunately, the issue of the action of ascorbic acid against uropathogens growing in the presence of urea had not been raised before. Our research is the first one presented in the literature. However, data regarding no effects of AA supplementation in the relief of symptoms of febrile upper urinary tract infection in children [15] indirectly confirm our findings about ineffectiveness of AA action under physiological conditions.

As early as 1936, German scientist J. von Gagyi [42] hypothesized that the antimicrobial properties of ascorbic acid resulted from its pH-lowering effect. Decades later, Robert Axelrod [43] stated that the AA action should be attributed to preventing the alkalization of urine by inhibiting the urease activity, rather than producing an acid urine. More recent data contest the effective action of AA (100–1000 mg) in acidifying urine of spinal cord injury patients [16], calcium-stone-forming patients [13,17], and healthy volunteers [13] and pregnant women [14]. What is important is that no bacteremia was found in any of these studies. On the other hand, research by Borran et al. [18] focused on the influence of AA on bacteriuria in kidney transplant patients and proved the effective action of a single dose of AA (70 mg kg^−1^) against the multiplication of bacteria. Moreover, Noureldin et al. [44] showed a significant decrease in the urinary pH value (from 7.6 to 6.9) of 24 patients with recurrent urolithiasis after a 1000 mg daily dose of AA. Another urine-acidifying agent is L-methionine. Although this amino acid is recommended by the EAU Guidelines on Urolithiasis for the treatment of infectious stones, its urine-acidifying properties appear to be as effective as for ascorbic acid [45]. As reported by Jacobs et al. [46], the daily intake of 1500 mg L-methionine significantly reduced urinary pH to the values of 6.0–6.2. Siener et al. [47] confirmed this range during studies carried out on 12 healthy volunteers. The data published by Sabiote et al. [48] showing that one 500 mg tablet of L-methionine every 12 h allowed maintaining urinary pH from 5.0 to 5.5 for 2 months seem to be promising in the field of UTI prevention.

It is known that ascorbic acid, a highly unstable molecule in aqueous solutions, is a donor of hydrogen ions, and in this way reduces urine pH [49]. In this study, we demonstrated a progressive decrease in the AA concentration with persistently acidic pH (5.39) of abiotic control (without *P. mirabilis* cells). The low pH value persisting over time proved that ascorbic acid can be an effective urine acidifier, provided that it is sterile. Therefore, it could be useful in the prevention of recurrent UTIs, rather than in their treatment, when uropathogens (mostly urease-positive) would alkalize urine by secreted ammonia. In our studies, a rapid alkalization of the ATCC 29906 and C12 cultures was noticed, despite an initial acidification of modified TSB medium by AA. Ascorbic acid does not seem to be a proper agent for the eradication of urease-producing bacteria. On the other hand, ascorbic acid has been found to have inhibitory effect on urease activity [50]. AA supplementation limited the urease activity of the *P. mirabilis* ATCC 29906 cells by 21%. Krajewska and Brindel [51] explained that AA inactivates urease by denaturation caused by lowered pH. However, this mechanism of action does not seem to be an adequate explanation for our results, due to the fact that the uropathogenic cells with decreased urease activity grew in an alkaline environment. Interestingly, the combined activity of AA and aminoglycosides stimulated urease activity. Overproduction of ammonia, which could contribute to the precipitation of struvite and apatite crystals and urine flow blockages [52], does not encourage the supplementation of ascorbic acid during aminoglycoside treatment. Interestingly, higher pH values of the *P. mirabilis* cultures did not correspond to increased levels of urease activity. According to Agha et al. [53], enhancement of urease activity does not have to be strictly a function of the pH value. The alkalization of *P. mirabilis* cultures could also be related to decomposition of peptones available in TSB medium.

Combination of antibacterial drugs with ascorbic acid to enhance antibiotic activity was tested against a broad spectrum of uropathogenic bacteria [24,34,54,55,56]. Most of these studies verified the synergistic effect of ascorbic acid with fluoroquinolones. AA has been reported as an agent enhancing the effectiveness of ciprofloxacin (CIP) and ceftriaxone action in the treatment of CAUTIs induced by *Candida* sp., *Citrobacter* sp., *E. coli*, *Enterobacter* sp., and *S. aureus* [54]. Similarly, a combination of levofloxacin and ascorbic acid increased the reduction in biofilm formation by *E. coli*, *Klebsiella* sp., *Citrobacter* sp., *Enterobacter* sp., *Proteus* sp., and *Pseudomonas* sp. on catheter surfaces even by 80–100% [24]. Although the actions of ciprofloxacin, levofloxacin, and ceftriaxone combined with AA are attributed to synergistic effects, the mechanism of this synergy remains unclear. We also confirmed anti-biofilm properties of combined treatment with aminoglycosides and ascorbic acid, which reduced catheter colonization by the *P. mirabilis* C12 cells. On the other hand, the research recently published by Kwiecińska-Piróg et al. [34] proved an increased *P. mirabilis* biofilm production during a simultaneous supplementation of the culture medium with AA and aminoglycosides or fluoroquinolones. Unfortunately, contradictory results available in literature, as well as insufficient knowledge on the interaction between drugs and ascorbic acid molecules, do not allow for an unambiguous answer as to whether combined treatment can be recommended to remove such a complicated structure as urological biofilm.

The validity of the concomitant use of ascorbic acid and antibiotics in the treatment of UTIs also seems to be questionable. Our results show that AA significantly decreased the effectiveness of aminoglycoside treatment against planktonic *P. mirabilis* cells. Similarly, Verghese et al. [55] questioned the effectiveness of the synergistic action of AA and CIP presenting no significant changes in optical densities of cultures of 50 uropathogenic isolates of *E. coli*. A probable cause of this phenomenon could be protection of bacterial cells against ciprofloxacin-induced cytotoxicity through the presence of AA [56]. These reports point to the activity of ascorbic acid as an antioxidant.

Phospholipids are the main constituents of cell membranes. Recently, the organization and function of anionic PLs in bacteria are gaining increasingly more attention. It has been suggested that PLs play an important role in forming biofilms, antibiotic susceptibility, and adaptation to osmotic stress or decreased pH [57]. On the other hand, modifications in the phospholipid profile could be induced by toxic substances. Such substances are positively charged aminoglycosides binding to the negatively charged PLs [58]. Moreover, it has been indicated that, despite their hydrophilic nature, aminoglycosides could be adsorbed onto the head groups of acidic phospholipids of the membrane bilayer and then diffuse laterally into the central cavity [59]. These data indicate that changes in the PL profile of bacteria treated with aminoglycosides are highly probable. Unfortunately, only a few reports regarding this issue are available. Bermingham et al. [60] proved that such a low concentration of streptomycin as 10 μg mL^−1^ causes a significant increase in the PE levels in *S. marcescens* cells. However, in a subsequent paper, the authors [61] excluded the importance of PE levels for *E. coli* resistance to streptomycin. In turn, Hussein et al. [62] showed significant perturbations of phospholipids levels induced by amikacin in the *P. aeruginosa* FADDI-PA111 strain. We also described strong modifications in the *P. mirabilis* PL profile. Amikacin and gentamicin altered the membrane composition of both the planktonic and adherent cells of the ATCC 29906 and C12 strains. PE 32:1, PE 36:0, PG 30:0, PG 30:2, and PG 33:1 were indicated as possible biomarkers. Their quantitative changes could have been related either with aminoglycoside toxicity or the mechanism of *P. mirabilis* resistance to these antibiotics. Tao et al. [63] indicated that PE 32:1, a lipid of *A. baumanni,* was upregulated in response to colistin, while PE 36:0 was shown by Palusińska-Szysz et al. [64] as a lipid whose level could correspond to the sensitivity of *L. micdadei* to the human LL-37 peptide. Although little information about membrane interactions of non-membrane-targeting antibiotics (especially those concerning membrane lipids) is available in the literature, recently a report was published excluding membrane damage, such as membrane disruption or pore formation, by aminoglycosides, macrolides, and fluoroquinolones [65].

Fatty acids, which are structurally phospholipids tails, are one of the most dynamic components of bacterial cells. They are often replaced and modified in lipids in response to many factors. Due to these abilities, bacteria can easily adapt to changing environmental conditions [66]. Therefore, some of them are proposed as biomarkers of either toxicity or resistance to the substances such as monochlorophenols [67], polycyclic aromatic hydrocarbons [68], organic solvents [69], and even antibiotics [61]. In the paper by Hussein et al. [62], significant modifications of FA composition in the *P. aeruginosa* FADDI-PA111 strain treated with amikacin were indicated. In our studies, we showed the aminoglycoside-modified *P. mirabilis* FA profile. However, alternations were more noticeable in the planktonic rather than in the adherent ATCC 29906 and C12 cells. We supposed that this was related to the 24 h biofilm structure and impeded penetration of cells by antibiotics. However, several FAs: *cis* C16:1, *cy* C17:0, *cis* C18:2, and *cis* C18:1 were indicated as possible biomarkers of aminoglycoside toxicity or *P. mirabilis* resistance to these antibiotics. The literature data suggest that *cis* C16:1 and *cis* C18:1 could be involved in the adaptive mechanism of *P. putida* in response to toluene [70] or in the resistance mechanism of *E. coli* against streptomycin [61]. Moreover, C16:1 and *cy* C17:0 could be related with the resistance of *E. faecalis* to cationic antimicrobial peptides [71], while C16:1 and *cy* C17:0 could be involved in the resistance of *E. coli* to tetracycline [72]. The FAs mentioned above seem to be more noticeable biomarkers of toxicity or drug resistance than PLs.

Despite frequent reports on antibacterial [19,20] and anti-biofilm [21,22,23,24] properties of ascorbic acid, none of them present the AA activity against membranes. In our studies, ascorbic acid only slightly changed the PL and FA profiles of the uropathogenic *P. mirabilis* cells. Our experience shows that the drastic alternations in the lipid composition of microorganisms are usually induced by highly toxic compounds [73,74,75]. Therefore, we suppose that the mechanism of the antibacterial effect of AA might not be caused by damage to microbial membranes. In the paper by Słaba et al. [76], the authors showed no significant influence of 1mM ascorbic acid on the *P. marquandii* FA profile, confirming our assumptions.

Oxidative stress causes cellular damage, not only to membranes, but also to proteins and nucleic acids. It seems to be an interesting direction of research on the antimicrobial effect of ascorbic acid, taking into account its pro-oxidative activity. This activity of AA is a reduction of Fe to Fe^2+^, which reacts with O_2_ and in the Fenton reaction leads to ROS formation, including H_2_O_2_, which in reaction with Fe^2+^ generates a highly reactive hydroxyl radical [77]. In our studies, ascorbic acid induced overproduction of HO• only in the C12 *P. mirabilis* cells adherent to the catheter. As early as 1988, Higson [78] reported that iron enhances ascorbate toxicity, and AA-mediated hydroxyl radical formation is responsible for damage to both acetylcholine and B esterases of *E. coli*. A decade later, McCormick et al. [79] confirmed the hypothesis that the enhancement of DNA damage and the increased mortality of *E. coli* mutants lacking superoxide dismutase are caused by AA-induced HO• generation. More recent research showed the effectiveness of a mixture consisting of cupric chloride, ascorbic acid, and sodium chloride in killing *B. globigii* spores [80], as well as cupric oxide nanoparticles combined with ascorbic acid targeting both *E. coli* and *S. aureus* [81]. In both cases, hydroxyl radical generation by AA was a probable mechanism of the biocidal action of these preparations. On the other hand, aminoglycosides—well known 30S ribosome inhibitors—also stimulate the formation of free radicals. After binding to the ribosome, the antibiotics promote NADH oxidation through the electron transport chain, which leads to superoxide (O_2_^•−^) formation. Subsequently, O_2_^•−^ damages Fe–S clusters, releasing the substrate for the Fenton reaction, which results in HO• formation [82]. In our studies, both AK and CN induced hydroxyl radical overproduction in the adherent cells of *P. mirabilis* C12 strain. The research team of Kohanski et al. [82] demonstrated that hydroxyl radical formation is an important component of the kanamycin-mediated killing of *E. coli*. Moreover, by applying an iron chelator in their studies, the authors proved the irrefutable importance of the metal availability for HO• generation induced by kanamycin. Moreover, Ye et al. [83] showed the relevance of alanine presence for kanamycin-induced ROS-mediated death of *E. tarda*, a wild-type multidrug-resistant strain. On the other hand, Kozłowski et al. [84] drew attention to the rate of hydroxyl radical formation by the Cu^2+^–kanamycin A complex influenced by various pH values. The gentamicin–Cu(II) complex is also a recognized catalyst of hydroxyl radicals formation [85]. Although both aminoglycosides and ascorbic acid generate overproduction of hydroxyl radicals, the combined activity of aminoglycosides and AA resulted in decreased levels of HO• in adherent cells of the *P. mirabilis* C12 strain, compared to the monotherapies. It could be associated with non-enzymatic reduction of hydroxyl radicals by AA, which took a single H atom from the antioxidant molecule, oxidizing it to monodehydroascorbate [49]. Keithahn and Lerchl [86] experimentally confirmed the ability of ascorbic acid to scavenge HO•, while Koskimäki et al. [87] mentioned that this antioxidant protects bacteria from this free radical. Nevertheless, dependence on the obtained results should be insightfully explored.

Membrane permeability is a key property for effective delivery of drugs to intracellular targets. Low membrane permeability often leads to insufficient effectiveness of in vivo treatment [88]. Aminoglycoside-mediated enhancement of membrane permeability was firstly confirmed in liposome-based research [89,90]. Subsequent studies were focused on microbial models. Hancock et al. [91] showed that gentamicin caused increased permeability of *E. coli* membrane for the hydrophobic fluorescent compound 1-N-phenyl-naphthylamine, while Bruni and Kralj [92] demonstrated a kanamycin-induced increase in *E. coli* membrane permeability for PI. In our studies, we observed the opposite results. Both amikacin and gentamicin caused a reduction in membrane permeability of the planktonic *P. mirabilis* cells. It is the most likely mechanism of resistance of the uropathogenic cells to the aminoglycosides. This result leads us to conclude that modifications of the lipid composition and the indicated PL and FA biomarkers described in this paper are related to the resistance mechanisms of the *P. mirabilis* ATCC and C12 strains to aminoglycosides, rather than to the toxic effect of these antibiotics on the bacteria. Lower membrane permeability is one of the three identified modes of bacterial resistance to aminoglycosides [11]. Maloney et al. [93] recognized this mechanism in an amikacin-resistant *P. aeruginosa* strain. Interestingly, even more radical limitation in the *P. mirabilis* membrane permeability was observed in the presence of ascorbic acid (both in the monotherapy and in combination with aminoglycosides). Słaba et al. [76] did not indicate a statistically significant impact of AA on *P. marquandii* membrane permeability. According to the experimental data by Hannesschlaeger and Pohl [94], membranes have surprisingly low permeability for ascorbate molecules. Therefore, stronger sealing of *P. mirabilis* membranes in response to AA is even more surprising. It seems that the uropathogenic cells defend themselves by limiting their membrane permeability not only against aminoglycosides but also against ascorbic acid. This suggests that ascorbic acid could negatively affect the *P. mirabilis* cells. However, we have not been able to discover this mechanism.

This study had two strengths. The first one was use of the concentrations of ascorbic acid, aminoglycosides, and urea that are achievable in the urinary tract and may reflect physiological conditions. This approach brings the research closer to clinical trials and the potential applicability of the obtained results or noted dependencies in future. The second strength was a diverse range of techniques (from fluorescent to chromatographic) involved in searching for the molecular mechanism of AA, which increased the chances for resolving a dispute over the legitimacy of the ascorbic acid application. On the other hand, the study had two limitations. The first one was the use of the TSB medium with urea instead of synthetic urine, whose composition more closely reflects physiological urine. This decision was made for fear of an insufficient number of cells for research due to poor bacterial growth on synthetic urine. The second limitation was due to the studies being carried out on bacterial colonization of the whole catheter surface instead of only its channel. We assumed that studies performed only on inner surfaces of the catheter would be technically difficult and would expose the obtained values to a greater dispersion, limiting their statistical significance.

## 4. Materials and Methods

### 4.1. Microorganisms and Growth Conditions

In this study, two uropathogenic *P. mirabilis* strains were applied. The C12 strain was obtained from the Department of Biology of Bacteria (University of Lodz, Lodz, Poland), while the reference strain ATCC 29906 was purchased from the American Type Culture Collection (Manassas, VA, USA). Our previous studies were carried out on these strains [25].

The *P. mirabilis* strains were stored deep-frozen (−80 °C) in the presence of DMSO (Avantor Performance Materials Poland S.A, Gliwice, Poland). The bacteria were grown on agar nutrient plates with the addition of 0.1% phenol (Avantor Performance Materials Poland S.A, Gliwice, Poland) to inhibit swarm colony expansion. Their colonies were used to inoculate tryptic soy broth (TSB, BioMaxima S.A., Lublin, Poland) modified by the addition of 9.3 g L^−1^ urea (Chempur, Piekary Slaskie, Poland). The applied concentration of urea was the minimal level found in human urine [36]. Twenty-four-hour pre-cultures, adjusted to an optical density of 5 on the McFarland scale, were used to inoculate the bacterial cultures (10%) cultivated with or without the presence of the following substances (Merck, Darmstadt, Germany): amikacin (563 mg L^−1^), gentamicin (600 mg L^−1^), ascorbic acid (3.4 g L^−1^). The above concentrations corresponded to those achievable in urine after a single 250 mg IM dose of amikacin [95], a single IM injection of 160 mg of gentamicin [96], or a single 1000 mg oral dose of ascorbic acid [35]. The bacterial cultures were incubated for another 24 h at 37 °C. Samples were collected after 0, 6, and 24 h.

Adherent cells were obtained in the following way. Sterile 0.5 cm pieces of the silicone-coated catheter (14Fr/ch 30 mL/cc 4.7 mm, Cezetel-Poznan, Poznan, Poland) were introduced into the *P. mirabilis* cultures and cultivated as described above. Samples were collected after 6 and 24 h. For analyses, unattached cells were washed away from the catheter surface by sterile distilled water. Then, the adherent cells were detached from the silicone through 20-min sonication in an ultrasonic cleaner (Ulsonix Cleaning Instruments, Berlin, Germany), followed by 2 min vortexing (IKA vortex shaker; IKA Poland Sp. z o.o., Warsaw, Poland) and finally 15 min centrifugation at 4500 rpm (Heraeus Multifuge 3S-R Centrifuge; Kendro, Osterode, Germany). The uropathogenic cells prepared in this way were used in lipidomic and fluorescence studies.

### 4.2. Growth and Culture pH Values and Urease Activity of P. mirabilis

An optical density (OD) of the bacterial cultures obtained according to Section 4.1 was established spectrophotometrically at λ = 550 using a microplate reader (Multiskan EX, LabSystem, Cracow, Poland). pH values of these cultures were measured using a pH-meter (Elmetron, Zabrze, Poland).

Urease activity of the *P. mirabilis* cells was evaluated according to Weatherburn’s method [97] with a few modifications. Briefly, 1 µL of the bacterial culture was suspended at 100 µL of a phenolic reagent (1 mL of phenol, 5 mg of sodium nitroprusside (Merck, Darmstadt, Germany), bidistilled H_2_O up to 100 mL) and 100 µL of hypochlorite reagent (10 mL of sodium hypochlorite (Avantor Performance Materials Poland S.A, Gliwice, Poland), 500 mg of sodium hydroxide (Avantor Performance Materials Poland S.A, Gliwice, Poland), bidistilled H_2_O up to 500 mL). After mixing of the well content on a 96-well plate (Merck, Darmstadt, Germany) and 30 min sample incubation at 37 °C, A_620_ was measured with the use of a microplate reader. The amount of ammonia (µg mL^−1^) released during urease-mediated decomposition of urea was appointed from the standard curve (R^2^ = 0.9727). The urease activity was a concentration of NH_3_ calculated per the amount of the enzyme. A method by Lowry et al. [98] was used for the measurement of the amount of total bacterial protein. First of all, 1 mL of the bacterial cultures was centrifuged in an Eppendorf tube (Eppendorf Poland Sp. z o.o., Warsaw, Poland) for 10 min at 14,000 rpm (Sigma 1-15 Centrifuge; SIGMA Laborzentrifugen GmbH, Osterode, Germany). Next, the cells were suspended in 100 µL of 2M NaOH and incubated overnight at 37 °C. A total of 10 µL of the samples was mixed with 100 µL of C solution placed in the wells on a 96-well plate and then incubated at room temperature. The C solution was obtained by mixing 12.25 mL of an A solution (20 g of Na_2_CO_3_ (Avantor Performance Materials Poland S.A, Gliwice, Poland) and 0.2 g of KNaC_4_H_4_O_6_·4H_2_O (Avantor Performance Materials Poland S.A, Gliwice, Poland), bidistilled H_2_O up to 1 L) with 250 µL of a B solution (0.5 g CuSO_4_·5H_2_O (Merck, Darmstadt, Germany), bidistilled H_2_O up to 100 mL). After 20 min incubation, 10 µL of diluted (1:2 *v/v* in water) Folin reagent (Avantor Performance Materials Poland S.A, Gliwice, Poland) was added to the wells. The mixtures were incubated for another 30 min at room temperature. A_550_ was measured using a microplate reader. The concentration of a total bacterial protein (mg mL^−1^) was estimated from the standard curve (R^2^ = 0.9606). The urease activity is given in units of µg NH_3_ (mg of protein)^−1^.

### 4.3. Catheter Surface Colonization by P. mirabilis

Coverage of the silicone catheter surface by uropathogenic cells was examined according to the MTT tetrazolium reduction assay described previously [25]. The catheter pieces devoid of unattached bacterial cells were placed in a 96-well plate containing a mixture consisting of 150 µL of modified TSB medium and 15 µL of MTT (3-(4,5-dimethylthiazol-2-yl)-2,5-diphenyltetrazolium bromide (Merck, Darmstadt, Germany) solution (5 mg mL^−1^ in phosphate-buffered saline) (PBS; Thermo Fisher Scientific, Waltham, MA, USA)). After 30 min incubation in a moist chamber at 37 °C, the catheter pieces were transferred into new wells containing a mixture consisting of 150 µL of DMSO and 25 µL of glycine buffer (Merck, Darmstadt, Germany). The process of dissolving formazan crystals took 30 min. After incubation, the catheter pieces were removed, and color intensity was measured spectrophotometrically at λ = 550 using a microplate reader.

### 4.4. HPLC−MS^2^ Methods for Quantitative Analyses of AK, CN, and AA in the Post-Culture Supernatants

Aminoglycosides were determined quantitatively in the examined samples by the modified procedures of Chan et al. [99] for AK and Sun et al. [100] for CN. Flow injection analysis (FIA) was used. The FIA–LC–MS^2^ analyses were performed on an Agilent 1200 LC system (Santa Clara, CA, USA) coupled with a 4500 QTRAP mass spectrometer (Sciex, Framingham, MA, USA). The mobile phase consisted of an ultrapure water/methanol (70:30) (J.T. Baker Chemical Company, Deventer, the Netherlands) mixture with the addition of 5 mM ammonium formate (Merck, Darmstadt, Germany) at a constant flow of 1 mL min^−1^. The mass detector was set to the positive ionization, multiple reaction monitoring (MRM) mode with an electrospray ionization (ESI) ion source. The optimized ion source parameters were as follows: curtain gas (CUR), 25; ionization voltage (IS), 5500 V; temperature, 600 °C. The MRM pair was m/z 586.24–163.1 for AK, while for CN components 478.32–139.1, 464.31–160.1, and 450.33–322.3 were applied. AA was analyzed using the Agilent 1200 HPLC system according to the method described by Słaba et al. [101]. Chromatographic separation was performed using a Kinetex C18 column ((50 mm × 2.1 mm, particle size: 5 μm; Phenomenex, Torrance, CA, USA) column temperature 37 °C, injection volume 10 µL). The eluents used were composed of water (A) and methanol (B), supplemented with 5 mM ammonium formate. The solvent was eluted at a constant flow rate of 500 µL min^−1^, starting with 80% of eluent A for 1 min, and then decreased to 50% of eluent A, which was maintained for 2 min. The initial conditions were restored for a further 2 min. The MS^2^ detection was performed using the MRM mode in the negative ionization. The optimized parameters of the electrospray ionization ion source were as follows: CUR, 25; IS, −4500 V; temperature, 500 °C; nebulizer gas (GS1), 40 and turbo gas (GS2), 60. The monitored MRM pairs were m/z 175–115 and 175–71 for AA.

### 4.5. Bacterial Lipid Extraction Procedure

*P. mirabilis* phospholipids were extracted and partitioned simultaneously in the Bligh and Dyer [102] method. Glass beads and 500 µL of methanol (Chempur, Piekary Slaskie, Poland) were added to washed cells, obtained according to the method described in Section 4.1. The cells were disintegrated 3 times for 20 s at 20 m s^−1^ using a ball mill (FastPrep-24, MP-Biomedicals, Santa Ana, CA, USA). The homogenate was transferred into a new Eppendorf tube containing 500 µL of chloroform (Chempur, Piekary Slaskie, Poland) and 400 µL of distilled water. The mixture was vortexed for 3 min and centrifuged for 5 min at 3000 rpm. The bottom layer was placed into a new Eppendorf tube and evaporated to dryness. Lipid extracts were dissolved in ultrapure methanol and stored at −80 °C.

### 4.6. HPLC–MS^2^ Method for Determination of P. mirabilis PLs

The lipids were determined according to our method described earlier [25] using an ExionLC AC (Sciex, Framingham, MA, USA) UHPLC system and a 4500 Q-TRAP mass spectrometer equipped with an ESI source. A total of 10 µL of lipid extract was injected onto an Eclipse XDB-C18, 50 mm × 4.6 mm, 1.8 µm column (Agilent, Santa Clara, CA, USA), heated at 40 °C, with the flow rate of 500 μL min^−1^. Ultrapure water (A) and methanol (B) were applied as a mobile phase, with both containing 5 mM ammonium formate. The solvent gradient was initiated at 70% B and, after 0.25 min, increased to 100% B for 1 min; then, it was maintained at 100% B for 6 min before being returning to the initial solvent composition over 2 min. The data analysis was conducted with Analyst v1.6.3 software (Sciex, Framingham, MA, USA). The phospholipids were determined qualitatively using an MRM method with the following standards for each class: phosphatidylethanolamine (PE 14:0/14:0; Merck, Darmstadt, Germany) and phosphatidylglycerol (PG 14:0/14:0; Merck, Darmstadt, Germany).

### 4.7. Bacterial Fatty Acid Isolation Procedure

Lipids isolated from *P. mirabilis* cells according to the method described in Section 4.5 were transformed to fatty acid methyl esters by the slightly modified procedure of Ichihara and Fukubayashi [103]. Briefly, the lipid extract dissolved in 750 µL of methanol was transferred into an Eppendorf tube. Then, two of the following reagents were added: 100 µL of toluene (Avantor Performance Materials Poland S.A, Gliwice, Poland) and 150 µL of 8% HCl (Avantor Performance Materials Poland S.A, Gliwice, Poland). The samples were vortexed for 3 min and incubated at 45 °C overnight. The next day, 500 µL of ultrapure hexane (J.T. Baker Chemical Company, Deventer, the Netherlands) and 500 µL of deionized water were added to the cooled samples. The Eppendorf tube content was vortexed for 3 min. Finally, a minimum of 300 µL of the upper layer was transferred to an insert of a chromatographic vial (Supelco Inc., Bellefonte, PA, USA).

### 4.8. GC–MS Method for P. mirabilis Fatty Acid Identification

The determination of fatty acid methyl esters was performed using our previously described method [25] with an Agilent Model 7890 gas chromatograph, equipped with a 5975C Mass Detector (Santa Clara, CA, USA). The separation was carried out using a capillary column HP 5 MS methyl polysiloxane (30 m × 0.25 mm i.d. × 0.25 mm ft, Agilent, Santa Clara, CA, USA). The column temperature was maintained at 60 °C for 3 min, then increased to 212 °C at the rate of 6 °C min^−1^, followed by an increase to 245 °C at the rate of 2°C min^−1^, and finally to 280 °C at the rate of 20 °C min^−1^. The column temperature was maintained at 280 °C for 5 min. Helium was used as a carrier gas at a flow rate of 1 mL min^−1^. The injection port temperature was 250 °C. Split injection was employed. The identity of fatty acid methyl esters in the samples was confirmed by the retention time and abundance of quantification ions in the authentic standards (Merck, Darmstadt, Germany).

### 4.9. Fluorescent Estimation of HO• Level

HO• production was examined with a cell-permeable, oxidation-sensitive fluorescent probe DCF using the modified method described by Słaba et al. [101]. Firstly, the bacterial cells obtained according to the method described in Section 4.1 were washed twice in PBS. Then, 10 µL of H_2_DCFDA (Merck, Darmstadt, Germany) solution (2 mg mL^−1^ in DMSO) was added to the cells suspended in 1 mL of PBS. The cells were labeled with H_2_DCFDA for 15 min in the dark at room temperature. The samples were centrifuged twice (for 10 min at 12,000 rpm) and washed in 1 mL of PBS removing residues of fluorophore. Then, H_2_DCFDA-labeled cells were suspended in 200 µL of the buffer and placed in a 96-well plate. Red fluorescence intensity of DCF (formed after H_2_DCFDA oxidation by ROS) was measured with a FLUOstar Omega spectrofluorometer (BMG Labtech, Ortenberg, Germany) with the following parameters: λex = 485–12 nm, λem = 520 nm, gain = 1000, target 70%. Followed by fluorescence measurements, optical densities of these samples were estimated according to the method described in Section 4.2. The results are shown as the ratio of the fluorescence intensity to the OD value.

### 4.10. Fluorescent Studies on the Bacterial Membrane Permeability

The membrane integrity of the uropathogens was examined using propidium iodide, the most frequently used fluorescence indicator for cell viability in terms of membrane permeability. The procedure had been previously described [75]. *P. mirabilis* cells cultivated according to the method described in Section 4.1 were washed twice in PBS. Next, 2 µL of PI (Thermo Fisher Scientific, Warsaw, Poland) solution (1 mg mL^−1^ in H_2_O) was added to the bacterial cells suspended in 1 mL of PBS. The cells were labeled with PI for 5 min in the dark at room temperature. The fluorophore residues were removed by alternating centrifugation (for 10 min at 12,000 rpm) and washing in 1 mL of PBS. Finally, PI-labeled cells were suspended in 200 µL of the buffer and placed in a 96-well plate. The red fluorescence intensity of PI intercalated to bacterial DNA was measured with a FLUOstar Omega spectrofluorometer with the following parameters: λex = 540–10, λem = 630–10, gain = 1000, target 70%. Followed by fluorescence measurements, optical densities of these samples were estimated according to the method described in Section 4.2. The results are shown as the ratio of the fluorescence intensity to the OD value.

### 4.11. Statistical Analyses

The experiments were conducted independently in triplicate. The amount of variability in dataset is provided as a standard deviation (±SD). Statistical significance was verified with Student’s *t*-test and Fisher’s tests. The analyses were performed using Excel 2016 (Microsoft Corporation, Redmond, WA, USA). Values at *p* ≤ 0.05 were considered significant.

## 5. Conclusions

Intensified multiplication of the aminoglycoside-resistant *P. mirabilis* cells as well as stimulation of their urease activity by ascorbic acid supplementation question the effectiveness of AA and aminoglycoside co-therapy. AA could be useful in the prevention of recurrent UTIs, rather than in their treatment, when urease-positive uropathogens alkalize urine by secreted ammonia. On the other hand, combined application of aminoglycoside and ascorbic acid has an anti-biofilm effect on the uropathogenic C12 strain. The mechanism of this action does not seem to result from perturbations of *P. mirabilis* cell membranes nor hydroxyl radical overproduction. Despite the fact that this study did not reveal the molecular basis of AA action, the limitation in *P. mirabilis* membrane permeability in response to the presence of ascorbic acid allows for the supposition that the molecular mechanism of AA activity does exist. The issue remains open and requires further investigation. In our opinion, the use of physiologically achievable AA and urea concentrations is crucial for the success of further research.

## Figures and Tables

**Table 1:** The results of the HPLC–MS^2^ quantitative analyses of amikacin, gentamicin, and ascorbic acid in abiotic and biotic cultures.

Strain	Culture	Quantified Compound	Concentration *		
			0 h	6 h	24 h
	Abiotic control	AK	559.81 ± 5.53		
		CN	601.32 ± 2.51		
		AA	3.40 ± 0.05	2.31 ± 0.01	0.020 ± 0.011
ATCC 29906	AA	AA	3.41 ± 0.40	1.30 ± 0.03	0.003 ± 0.003
	AK + AA	AA		0.98 ± 0.06	0.001 ± 0.001
	CN + AA	AA		0.58 ± 0.03	0.005 ± 0.002
C12	AA	AA	3.44 ± 0.18	1.97 ± 0.06	BDL **
	AK + AA	AA		0.37 ± 0.01	BDL **
	CN + AA	AA		1.16 ± 0.03	BDL **

* Concentrations are given in the following units: mg L^−1^ for amikacin (AK) and gentamicin (CN), g L^−1^ for ascorbic acid (AA). ** BDL—below the detection limit.

**Table 2:** Statistically significant changes in the PLs levels induced by AA, AK, or CN treatment in the planktonic and adherent cells of the *P. mirabilis* strains, determined by Student’s *t*-test.

	ATCC 29906 Strain						C12 Strain					
Phospholipid	Planktonic Cells			Adherent Cells			Planktonic Cells			Adherent Cells		
	AA	AK	CN	AA	AK	CN	AA	AK	CN	AA	AK	CN
PE 34:1		*	*					*	*			
PE 30:0		*						*				
PE 32:0		*	*					*	*			
PE 32:1		*	*		**	**		*	*		**	**
PE 32:2		*						*				
PE 33:1		*	*			**		*	*			**
PE 34:0		*	*		**			*	*		**	
PE 34:2		*						*				
PE 36:0		*	*		**	**		*	*		**	**
PE 36:2					**	**					**	**
PE 37:2			*						*			
PE 38:2												
PG 29:2					**						**	
PG 30:0		*	*	**	**	**		*	*	**	**	**
PG 30:2		*	*		**	**		*	*		**	**
PG 31:2		*	*					*	*			
PG 32:2		*						*				
PG 33:1		*	*		**	**		*	*		**	**

Legend: C—control, AA—ascorbic acid, AK—amikacin, CN—gentamicin. The analyses were performed relative to the contents of PLs in the non-treated uropathogenic cells. The increased lipid levels are marked in green, and the decreased ones in red. Common trends in phospholipid quantitative changes in both examined strains are marked by (*) and (**) for the planktonic and adherent cells, respectively.

**Table 3:** Statistically significant changes in the PLs levels induced by combined treatment with ascorbic acid and amikacin or gentamicin in the planktonic and adherent cells of the *P. mirabilis* strains, determined by Student’s *t*-test.

Phospholipid	ATCC 29906 Strain				C12 Strain			
	Planktonic Cells		Adherent Cells		Planktonic Cells		Adherent Cells	
	AK +AA	CN +AA	AK +AA	CN +AA	AK +AA	CN +AA	AK +AA	CN +AA
PE 34:1								
PE 30:0				**				**
PE 32:0			**				**	
PE 32:1	*			**	*			**
PE 32:2		*				*		
PE 33:1			**				**	
PE 34:0								
PE 34:2		*	**			*	**	
PE 36:0			**				**	
PE 36:2								
PE 37:2		*		**		*		**
PE 38:2	*				*			
PG 29:2								
PG 30:0								
PG 30:2			**				**	
PG 31:2								
PG 32:2		*		**		*		**
PG 33:1		*				*		

Legend: C—control, AA—ascorbic acid, AK—amikacin, CN—gentamicin. The analyses were performed relative to the contents of PLs in the uropathogenic cells treated with AK or CN. The increased lipid levels are marked in green, and the decreased ones in red. A common trend in phospholipids quantitative changes in both examined strains is marked by (*) and (**) for the planktonic and adherent cells, respectively.

**Table 4:** Statistically significant changes in the FAs levels induced by AA, AK, or CN treatment in the planktonic and adherent cells of the *P. mirabilis* strains, determined by Fisher’s test.

	ATCC 29906 Strain						C12 Strain					
Fatty Acid	Planktonic Cells			Adherent Cells			Planktonic Cells			Adherent Cells		
	AA	AK	CN	AA	AK	CN	AA	AK	CN	AA	AK	CN
C14:0												
*cis* C16:1		*	*					*	*			
C16:0												
*cy* C17:0		*	*					*	*			
*cis* C18:2		*	*					*	*			
*cis* C18:1		*	*					*	*			
C18:0												

Legend: C—control, AA—ascorbic acid, AK—amikacin, CN—gentamicin. The analyses were performed relative to the contents of FAs in the non-treated uropathogenic cells. The increased lipid levels are marked in green, and the decreased ones in red. A common trend in fatty acid quantitative changes in both examined strains is marked by (*) for the planktonic cells.

**Table 5:** Statistically significant changes in the FA levels induced by combined treatment with ascorbic acid and amikacin or gentamicin in the planktonic and adherent cells of the *P. mirabilis* strains, determined by Fisher’s test.

Fatty Acid	ATCC 29906 Strain				C12 Strain			
	Planktonic Cells		Adherent Cells		Planktonic Cells		Adherent Cells	
	AK +AA	CN +AA	AK +AA	CN +AA	AK +AA	CN +AA	AK +AA	CN +AA
C14:0								
*cis* C16:1								
C16:0								
*cy* C17:0								
*cis* C18:2								
*cis* C18:1								
C18:0								

Legend: C—control, AA—ascorbic acid, AK—amikacin, CN—gentamicin. The analyses were performed relative to the contents of FAs in the uropathogenic cells treated with AK or CN. The increased fatty acids levels are marked in green, and the decreased ones in red.

## Data Availability

Not applicable.

## Supplementary Materials

The following supporting information can be downloaded at: https://www.mdpi.com/article/10.3390/ijms232113069/s1.
