# Supplementary material for: Combined Application of Aminoglycosides and Ascorbic Acid in the Elimination of Proteus mirabilis Rods Responsible for Causing Catheter-Associated Urinary Tract Infections (CAUTIs)—A Molecular Approach
Source: Int J Mol Sci. 2022 Oct 28;23(21):13069. doi: 10.3390/ijms232113069 (PMC9659235; doi:10.3390/ijms232113069)
Supplement: Supplementary file 1 [file ijms-23-13069-s001.zip › ijms-1962623-supplementary.pdf]

SUPPLEMENTARY MATERIAL

Combined Application of Aminoglycosides and Ascorbic Acid in the Elimination of *Proteus mirabilis* Rods Responsible for Causing Catheter-Associated Urinary Tract Infections (CAUTIs)—A Molecular Approach

Paulina Stolarek [1,*], Przemysław Bernat [2] and Antoni Różalski [1]

[1] Department of Biology of Bacteria, Faculty of Biology and Environmental Protection, University of Lodz, Banacha 12/16, 90-237 Lodz, Poland

[2] Department of Industrial Microbiology and Biotechnology, Faculty of Biology and Environmental Protection, University of Lodz, Banacha 12/16, 90-237 Lodz, Poland

* Correspondence: paulina.stolarek@biol.uni.lodz.pl; Tel.: +48-42-635-43-24; Fax: +48-42-665-58-18

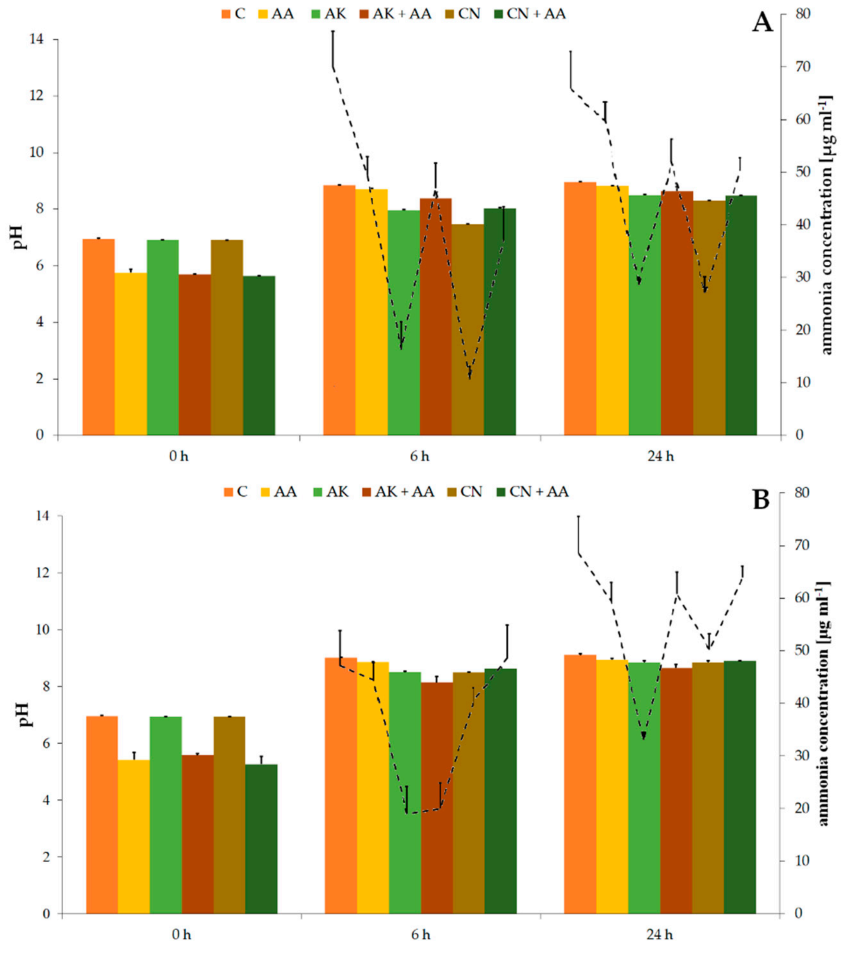

Figure S1. Changes in the pH values (column graphs) and ammonia levels (line graphs) in the *P. mirabilis* cultures incubated in the presence of the aminoglycosides or/and ascorbic acid. Legend: C – control, AA – ascorbic acid, AK – amikacin, CN – gentamicin.

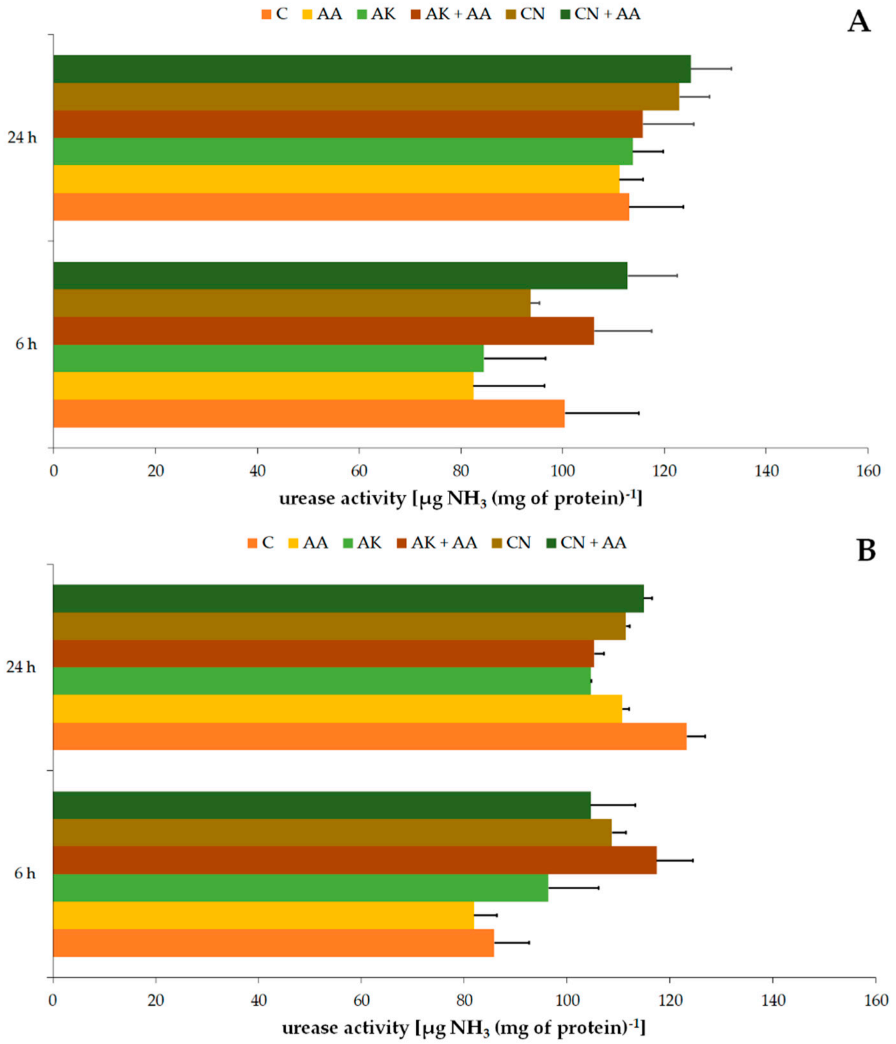

Figure S2. Differences in the urease activity of the *P. mirabilis* ATCC 29906 (**A**) and C12 (**B**) strains caused by the action of the aminoglycosides or/and ascorbic acid. Legend: C – control, AA – ascorbic acid, AK – amikacin, CN – gentamicin.
